# Migrant experiences of sexual and gender based violence: a critical interpretative synthesis

**DOI:** 10.1186/s12992-022-00860-2

**Published:** 2022-06-28

**Authors:** Sze Eng Tan, Katie Kuschminder

**Affiliations:** 1 UNU-MERIT / Maastricht University, Boschstraat 24, 6211 AX Maastricht, The Netherlands; 2 Department of Political Science, University of Amsterdam, Nieuwe Achtergracht 166, 1018 Amsterdam, WV Netherlands

**Keywords:** Migration, Gender-based violence, Sexual violence, Vulnerable, Worldwide, Critical interpretive synthesis

## Abstract

**Background:**

Gender based violence (GBV) is a critical issue and migrants are at higher risk of experiencing and being victimized by GBV. This critical interpretative synthesis (CIS) examines migrants experiences of GBV with a focus on different migrant groups and experiences at different stages of the migrant journey.

**Method:**

The guiding question of this review is: “how do migrants experience gender-based violence?” A total of 84 studies were included in the CIS, of which 67 peer-reviewed academic articles were selected from 2356 studies found on WebofScience, MedLINE, and ProQuest, and 17 relevant studies from the grey literature were selected from the time period 2011 to 2020. All final studies were reviewed and synthesized using a critical inductive approach to formulate the key results.

**Results:**

The results demonstrate a high prevalence of GBV amongst migrants, and in particular among vulnerable migrant groups such as forced migrants and irregular migrants, with an emerging focus on male victims. Findings of the CIS revealed three key themes: 1) Most GBV occurrences are rooted in unequal power dynamics; 2) Victims often live with long-lasting consequences that are worsened by their fear of disclosure and stigmatization; 3) There are differential understandings of victimhood across organizations, communities, and victims themselves. In order to support access, sampling, and methodological challenges in this field of research, this article also reports its findings on common risk-factors identified, consequences and coping mechanisms reported, protection policies targeting GBV, and finally, available databases and data collection methods.

**Conclusion:**

Further directions for research should be encouraged to move beyond prevalence reporting into identifying risk-factors and possible prevention in both sexes. In addition, more research on GBV experiences throughout migrants’ journeys, and coping mechanisms should be encouraged.

## Introduction

Gender-based violence (GBV) poses a significant health and judicial concern to societies worldwide, carrying devastating consequences for victims as well as their families, relations, and communities. Within the context of migration, GBV experiences are difficult to record, with research and support policies mainly relying on self-disclosure rates which differ across data collection methods [1]. Not only experiencing, but also being exposed to GBV can be distressing and socially stigmatizing, making it difficult to provide GBV-specific support to victims who prefer not to disclose their experiences [2]. Further, migrants with unstable residency status in the host countries experience economic, social, and institutional barriers in seeking judicial redress and support [3, 4]. The lack of pathways for these migrants to seek protection from GBV without fear of consequences such as deportation, detention, or ostracization from the community has a direct deterrence effect on the reporting of GBV experiences [5, 6].

This critical interpretive synthesis (CIS) has started from the question: How do migrants experience GBV? With the sub-questions of:

- How is GBV experienced by different types of migrants?
- When and where is GBV experienced by migrants, including at what stage of their migration cycle?

As a CIS, these questions have loosely guided the review. The aim of this article is to present the CIS results in order to first, understand current academic developments on this topic and second, critically synthesize and identify emerging themes within this field of study to lead to further theorization of the relationship between migration and GBV.

The first section of this article presents an overview of guiding definitions used in the article for GBV and migration. The second section details the CIS methodology used in this article. The third section presents a descriptive overview of the sources selected for inclusion in the CIS and the fourth presents the results of the CIS. The findings of this article are split into an overview of the prevalence and severity of GBV experienced by migrants, followed by three emerging themes: 1) Most GBV occurrences are rooted in unequal power dynamics; 2) Victims often live with long-lasting consequences that are worsened by their fear of disclosure and stigmatization; 3) There are differential understandings of victimhood across organizations, communities, and among victims themselves. The final sections of the article provide a discussion including limitations of the CIS and a summative conclusion.

## Background and definitions

Although there are many different definitions of GBV, the most commonly used definitions are provided by the The United Nations Entity for Gender Equality and the Empowerment of Women (UNWomen) and United Nations High Commissioner for Refugees (UNHCR). UNWomen defines GBV as:

“Gender-based violence (GBV) refers to harmful acts directed at an individual or a group of individuals based on their gender. It is rooted in gender inequality, the abuse of power and harmful norms” [7].

UNHCR follows a similar notion in its definition, but further elaborates that GBV is:

“An umbrella term for any harmful act that is perpetrated against a person’s will and that is based on socially ascribed (i.e. gender) differences between males and females. It includes acts that inflict physical, sexual or mental harm or suffering, threats of such acts, coercion, and other deprivations of liberty. These acts can occur in public or in private. (…) Acts of GBV include but are not limited to; purposeful denial of opportunities, intimate partner violence, trafficking for purposes of sexual exploitation, sexual abuses, forced child marriage, female genital mutilation, sexual servitude, and compelled transactional sex [8]”.

This article follows the UNHCR definition for GBV due to elaboration of the scope of GBV acts that it provides. In order to make a distinction across the levels of voluntariness in migration and thus vulnerability to GBV, common terminologies used are defined below in Table 1.

**Table 1:** IOM definitions used for identifying different migrant types

Migrant type	Definition	Source
Documented migrant	A migrant authorized to enter and to stay pursuant to the law of that State or to international agreements to which that State is a party and who is in possession of documents necessary to prove his or her regular status in the country.	International Organisation for Migration (IOM) [9]
Undocumented migrant	A non-national who enters or stays in a country without the appropriate documentation.	
Asylum-seeker	An individual who is seeking international protection. In countries with individualized procedures, an asylum seeker is someone whose claim has not yet been finally decided on by the country in which he or she has submitted it.	
Refugee (prima facie)	Persons recognized as refugees, by a State or the United Nations High Commissioner for Refugees, on the basis of objective criteria^a^ related to the circumstances in their country of origin, which justify a presumption that they meet the criteria of the applicable refugee definition.	
Family reunification (dependent) migrant	In the migration context, any person who is granted entry into a State for the purpose of family reunification on the basis of being supported by a “sponsor” with whom the individual has a proven family relationship.^b^	

^a^The Prima Facie recognition of refugee status is applied on the basis of apparent circumstances, whereby often, entire groups have been displaced and members can be considered as refugees individually, in absence of evidence to the contrary. The mentioned circumstances that qualifies one as a refugee is defined as per the 1951 Refugee convention (p.171)

^b^Although states differ in the type of family relations recognised for family reunification, they are most commonly spouses or children (below a specified age whereby they are still dependent on their parents) (p.44). However, it is known that some countries also grant entries for dependent (elderly) parents, and / or other relatives under specific considerations as per the country

## Methodology

### Critical interpretive synthesis (CIS) approach

Critical Interpretive Synthesis (CIS) was selected as the most suitable approach to this subject as it allows for integration of both quantitative and qualitative empirical studies into one review [10]. Given that this article seeks to understand the phenomenon of GBV in migration beyond its occurrence, qualitative studies provide a crucial insight into the severity, coping mechanisms, and human behavior of migrants when faced with (the risks of) GBV.

CIS is characterized by its interpretation and synthesis across literature, in which an inductive approach is applied to create an overarching understanding of the field. This is done by reviewing, comparing, and analyzing relevant literature for emerging themes and phenomena [11]. In the CIS approach, no well-defined research question is necessary at the onset of the process – rather, a fuzzy review question at the onset becomes consistently refined through the search and review process [12]. This iterative process incorporates flexibility in its search, sampling, and synthesis process which undoubtedly affects its findings. The lack of current CIS reviews on migrants’ experiences of GBV worldwide, along with the broad subject of GBV, makes CIS a suitable methodology for our research question. However, the iterative process in CIS also means that some parts of the process may not be reproducible, which this review acknowledges as its primary limitation. To this effect, we seek to maximize internal validity with more rigorously tracked and explicit reporting of procedures for literature search including that of grey literature, findings of empirical research, along with more elaboration on the derivation of concepts and conclusions from selected literature. As a result, a list of search terms, definitions guiding the ex/inclusion criteria, and a detailed figure presenting the review results are presented in the next section.

### Literature search and sampling

For academic literature, a search for relevant terms was conducted between 1st February 2021 to 22nd February 2021 on three major databases: WebofScience (all), MedLine, and ProQuest. Only peer-reviewed articles from between 2011 to 2020 were searched. We used a timeframe from 2011 to 2020 to get a robust overview of contemporary discourse in this field that emerged within the past decade. Furthermore, a preliminary search covering 2011 to 2020 confirmed that the timeframe yielded sufficient results to reflect on the research questions. Table 2 below lists the search strings used.

**Table 2:** Search terms and databases used for academic literature search

Search Terms		Database	Search Filter
Subjects	Themes / acts		
“Migrant” and: Transit; child; adolescent; young; female; male; asylum-seeker; refugee; trafficked; victim; economic; labor; marriage; MAR	“Sexual” or “Gender” and: abuse; violence; assault; rape; harassment; exploitation; transactional; forced; mutilation	• WebofScience • MedLINE • ProQuest	• Year: 2011–2020 • Academic articles only • Peer-reviewed

The inclusion criteria consisted of any English language articles published after 2011 on GBV and migration. Articles were considered relevant if they are original articles with empirical evidence, or if they carried theoretical, legislative, methodological contributions to the field of GBV and migration. As there is a large number of articles on the topic of GBV which intersect with or involve migrants as a secondary interest, a decision was made to select only articles that carry migrants as the primary subject in document titles and abstracts. Any migrant types and geographical locations were accepted during the search process in order to capture a wide set of lived experiences. We only considered papers centering on acts of sexual violence where; 1) it was perpetuated against a person’s will and/or, 2) is perpetrated based on socially ascribed differences between genders. This removed papers that discussed broader forms of sexual discrimination (eg: unequal access to reproductive health care, asexualisation of female domestic workers, and so on).

In line with most CIS reviews, grey literature was also considered for this review. This refers to documents, usually reports, working papers, or policy documents that have not necessarily gone through a peer-review process. Given the humanitarian nature of GBV as a topic, reports by international organizations and non-governmental organizations such as UNHCR, IOM, and MSF that are involved in the frontlines of migrant GBV prevention and support undoubtedly contain salient insights on the topic. The search for grey literature was informed by prior sampling and reviewing of academic literature, where relevant organizations and search terms were identified.

During the first phase of the search process, academic article titles were screened for any possible relevance and noted down. Reading of abstracts then refined the number of papers selected. In the second phase of the search, entire articles were carefully read and labelled for themes. Through the reading process, themes and phrases that commonly occurred were identified and added as search terms, repeating the process in Phase I until no new key terms emerged from reading. In this phase, knowledge was also developed on key organizational and institutional stakeholders in the field, which informed the grey literature search. The third and final phase was the backwards snowballing of references from the existing list of academic and grey literature. References were selected by reading, in order: the title, abstract, and the full paper for relevance. Replicated papers were excluded.

**Fig. 1:**
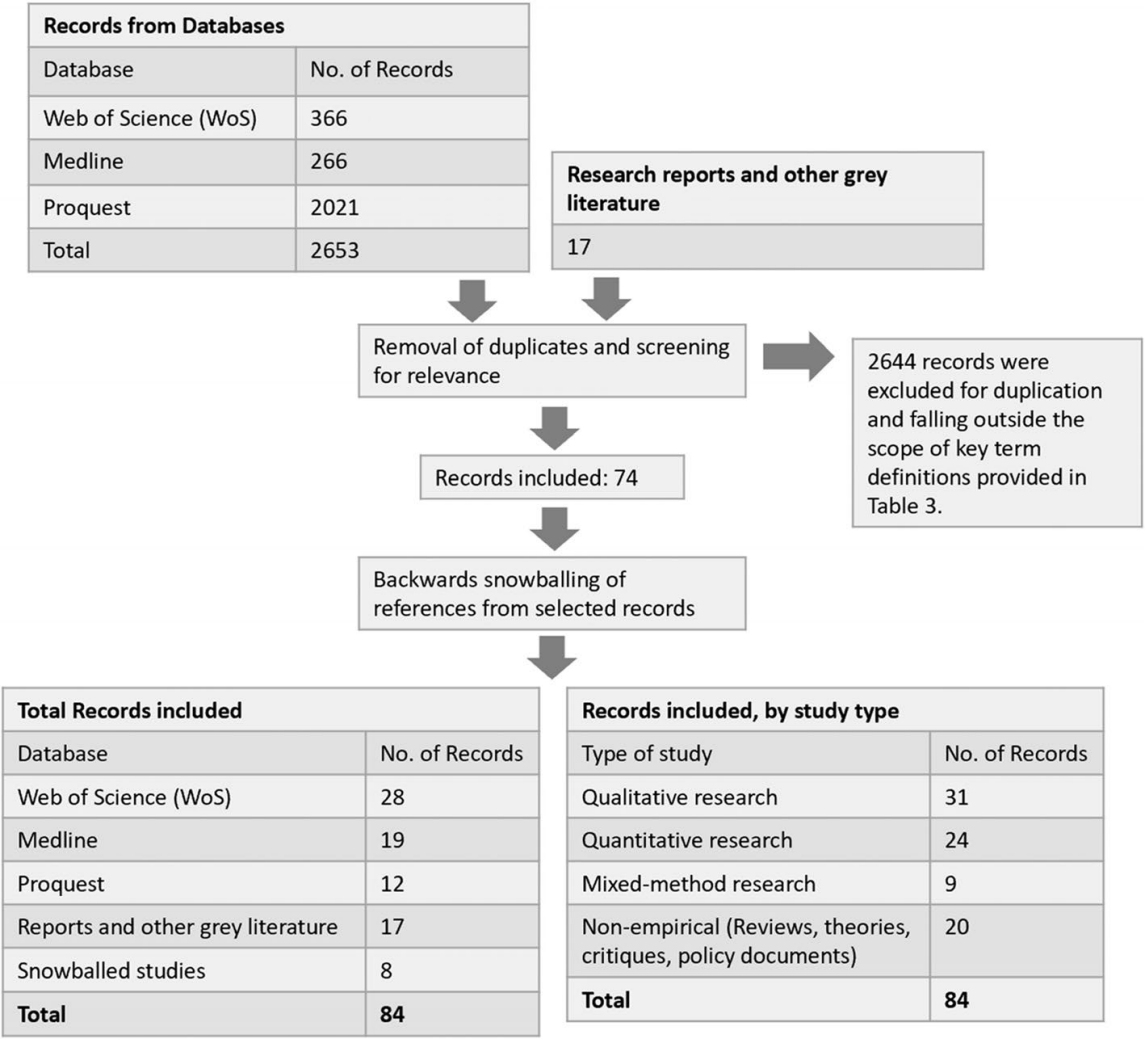
Review process and selected records

As GBV is largely characterized by differentiation between genders, the acts of violence in itself can overlap in definitions. During the process of sampling and reviewing papers, we have also identified several key terms and their established definitions which helped with further identification of literature. The definitions listed in Table 3 are those provided (ad verbatim) by the corresponding institutions. It is important to note that this list serves as guiding definitions found and subsequently used in the iterative search process – it is not an exhaustive or all-encompassing list for the types of sexual violence that individuals may experience. A total of 2653 journal articles were identified on migration and GBV. After the three phases of search, a total of 67 peer-reviewed journal articles and 17 grey literature documents were selected for this review. Most academic articles were taken from WebofScience (WoS) as it was the first database searched. Fewer papers were selected from subsequent databases as results were mostly repeated from WoS. The review process, initial search results and the number of final selected papers from each database can be seen in Fig. 1 below.

**Table 3:** Definition of Key Terms by United Nations (To appear at the end of Page 11)

Key terms	Definition	Reference
Victim	Persons who, individually or collectively, have suffered harm, including physical or mental injury, emotional suffering, economic loss or substantial impairment of their fundamental rights	United Nations General Assembly [13]
Violence against women	any act of gender-based violence that results in, or is likely to result in, physical, sexual or psychological harm or suffering to women, including threats of such acts, coercion or arbitrary deprivation of liberty, whether occurring in public or in private life	United Nations General Assembly [14]
Transactional Sex	The exchange of money, employment, goods or services for sex, including sexual favours other forms of humiliating, degrading or exploitative behaviour. This includes any exchange of assistance that is due to beneficiaries of assistance.	United Nations [15]
Trafficking of persons for sexual exploitation	The recruitment, transportation, transfer, harbouring or receipt of persons, by means of the threat or use of force or other forms of coercion, of abduction, of fraud, of deception, of the abuse of power or 8 of a position of vulnerability or of the giving or receiving of payments or benefits to achieve the consent of a person having control over another person, for the purpose of sexual exploitation	
Conflict-related sexual violence	Incidents or patterns of sexual violence – including rape, sexual slavery, forced prostitution, forced pregnancy, forced abortion, enforced sterilization, forced marriage and any other form of sexual violence of comparable gravity - perpetrated against women, men, girls or boys that is directly or indirectly linked (temporally, geographically or causally) to a conflict.	
Sexual Violence	Acts of a sexual nature against one or more persons or that cause such person or persons to engage in an act of a sexual nature by force, or by threat of force or coercion, such as that caused by fear of violence, duress, detention, psychological oppression or abuse of power, or by taking advantage of a coercive environment or such person’s or persons’ incapacity to give genuine consent.	
Sexual Abuse	Actual or threatened physical intrusion of a sexual nature, whether by force or under unequal or coercive conditions.	
Rape	Penetration – even if slightly – of any body part of a person who does not consent with a sexual organ and/or the invasion of the genital or anal opening of a person who does not consent with any object or body part.	
Sexual Assault	Sexual activity with another person who does not consent. It is a violation of bodily integrity and sexual autonomy and is broader than narrower conceptions of “rape”, especially because (a) it may be committed by other means than force or violence, and (b) it does not necessarily entail penetration.	
Exploitative relationship	A relationship that constitutes sexual exploitation, i.e. any actual or attempted abuse of a position of vulnerability, differential power or trust, for sexual purposes, including, but not limited to, profiting monetarily, socially or politically from the sexual exploitation of another.

## Overview of studies

Table 4 below shows the geographical coverage of the studies included in the review. As only papers in English were selected due to accessibility of databases and language knowledge, this review is arguably limited in geographic coverage. However, our methodology was unable to assess the degree of under-representation of non-English reporting regions.

**Table 4:** No. of studies selected, by region of GBV occurrence or data collection (excluding 4 policy documents and two review documents)

Region of GBV occurrence	No. of studies
European Union	25
Sub-Saharan Africa	23
Asia-Pacific	8
Middle East and North Africa	9
Americas	8
Balkan States	1
More than one region	8

Table 5 shows the number of studies focusing on different groups of migrants. The majority of studies were focused on forcibly displaced migrants. However, through the review we have identified that there is a lack of data on refugees living outside camps, asylum-seekers, and other irregular migrants.

**Table 5:** No. of studies selected, by migrant type (excluding 4 policy documents)

Primary focus	No. of studies
Asylum-seekers / Refugees	41
More than one group	21
Undocumented migrants	9
Minors	2
Regular / Labour migrants	3
Trafficking victims	1
Family reunification migrants	1
Internal migrants	2

### Existing statistics on quantitative data collection

Regarding existing quantitative data on GBV – UNHCR publishes microdata containing GBV-related variables in their Protection Monitoring data collected by fieldworkers [16]. These variables include prevalence of sexual harassment, exploitation, rape, and transactional sex. Similarly, the Mixed Migration Centre publishes regular trend reports with data collected on GBV prevalence and characteristics [17] – although the microdata is not yet available for viewing, an interactive data portal [18] has also been launched.^1^ The European Union (EU) agency for Fundamental Rights (FRA) collects and publishes data on violence against women within the EU, where survey data can be differentiated between immigrants and local residents [19]. For minors, United Nations Children’s Fund (UNICEF) collects and publishes data in situation reports on GBV and child migrants. UNICEF’s Multiple Indicator Cluster Surveys (MICS) include variables related to sexual harassment and violence from migrant children that can be useful in GBV research [20]. Within the Asia-Pacific region, The Economic and Social Commission for Asia and the Pacific (UN ESCAP) publishes data on prevalence of sexual violence within its data explorer – although this data does not focus on migrants specifically, it may be useful as a tool for comparison on migrants’ GBV prevalence in origin and host countries [21].

On trafficking, other than trafficking statistics published by The United Nations Office on Drugs and Crime (UNODC) [22] and IOM’s Counter Trafficking Data Portal [23], the Asian-Pacific Institute on Gender-Based Violence also publishes consolidated data related to GBV within the Asia-Pacific region [24]. The Global Slavery Index also consolidates indicators published by the International Labor Organization (ILO) and IOM on Global Estimates of Modern Slavery, which includes trafficking and abuses of (ir)regular migrants [25]. A non-exhaustive list of publicly available data source is shown in Table 6.

**Table 6:** Publicly available and known data on migration and GBV

Data source	Migrant type	Description
UNHCR Microdata, Protection monitoring	Refugees	Contains indicators for survival sex, experience with sexual harassment
UNHCR Global Focus – Thematic page on SGBV Prevention and Response	Refugees	Annual data on no. of reported GBV cases, percentage of rape survivors receiving post-exposure prophylaxis (PEP) for HIV within 72 hours of incidence, extent to which survivors receive support, and extent to which is active in GBV prevention
4Mi Trend Reports, Mixed Migration Center	Multiple	Interactive data portal (upcoming) and regular trend reports of which includes GBV
EU Agency for Fundamental Rights	Multiple	Violence against women within the EU
UNICEF Multiple Indicator Cluster Surveys (MICS)	Minors	Sexual harassment and violence
UN ESCAP	Multiple	Prevalence of sexual violence across countries
UNODC Global Report on Trafficking In Persons	Trafficking victims	Data and statistics on those trafficked for sexual exploitation
IOM Counter trafficking data collaborative	Trafficking victims	Data and statistics on those trafficked for sexual exploitation
Asian-Pacific Institute on Gender Based Violence	Multiple	Consolidated statistics from other sources within the Asia-Pacific region
Global Slavery Index	Multiple	Consolidated indicators from ILO and IOM on trafficking and abuse of migrants

### Participatory methods and qualitative research

It is evident that primarily qualitative methods have been used in previous studies. Given the sensitivity of the topic it is unsurprising that qualitative methods are used more frequently to build rapport and trust with migrants, as well as to provide space for them to fully explain their experiences and stories.

Among the studies reviewed in this article, the largest academically collected data sample was completed through community-based participatory research (CBPR) This method is encouraged as a way of establishing mutual trust and deeper understanding between researchers and respondents [26–28]. This is in line with conventional guidelines on GBV research methodologies [29].

Other methods for qualitative data collection within the studies included focus-group discussions and following of migrants’ journeys over a period of time to identify areas of vulnerabilities and structural violence. Qualitative studies found in this review had a mix of sample sizes, ranging from 25 to 223 respondents depending on the targeted population, with most studies including upwards of more than 50 respondents. Most qualitative studies were focused on the causes (risk-factors) and possible prevention of GBV through inputs from stakeholders. These studies make substantive contributions to informing understandings of GBV processes and possible interventions for prevention and treatment.

### Data collection techniques

Data on migration and GBV are difficult to collect due to the sensitivity of the topic, stigma attached to GBV and resulting low rates of self-disclosure. Evidence has shown that the gender of the interviewer or researcher should be taken into consideration, not merely for establishing trust with female respondents, but also in accessing female respondents within more patriarchal communities. Thambia, Chakraborty [30] reflected that there is a necessity for female researchers to develop coping mechanisms and to learn how to negotiate with gatekeeper violence when trying to access female migrants within male-dominated contexts.

Anonymous, self-administered surveys are considered an effective way to collect GBV data that bypasses the psychological need to deliver socially acceptable answers [1]. The ASIST-GBV, a form of anonymous self-administered GBV survey, has been used to screen for GBV within humanitarian settings [31]. The study by Roupetz, Garbern [32] elaborated on their measure of using a SenseMaker survey – a self-administrated survey that does not ask explicit questions on GBV but instead allow for narratives to allow GBV experiences to become apparent. These methods of data collection have been considered to be largely successful thus far by the cited studies.

## Results

The following section presents the findings from the CIS of the key themes represented in the studies. The first section presents an overview of the prevalence of GBV experiences of different types of migrants, where the experience occurred and at what stage in their migration journeys. This section is largely in response to our initial scoping of the study. The results are furthered by an inductive analysis presenting key themes emerging from the studies that have been synthesized. This includes: the interplay of power dynamics between victims and perpetrators, lasting consequences and the fear of disclosure, and differential understandings of victimhood.

## Prevalence and experiences of GBV

Across the studies, it is consistently observed that rates of victimization are first, higher for migrants than compared to local populations and second, significantly higher for vulnerable groups of migrants. The latter are defined as such due to their documentation status or individual characteristics (gender, sex, age-group, etc.,) which places them at higher-risk for GBV victimization. This section discusses the emerging focus on vulnerable migrant groups, children, and male victims.

### High prevalence among vulnerable migrants in destination and transit countries

Empirical studies consistently show high rates of victimization among undocumented migrants, asylum-seekers, and refugees both while in transit and within destination countries [26, 33–36]. In particular, a cross-sectional study by Oliveira, Keygnaert [34] conducted in European reception asylum facilities (EARF) across seven EU countries^2^ found that 50.1% of facility residents interviewed experienced at least one case of GBV in the year prior to the interview. Another study by Keygnaert, Vettenburg [37] in Belgium and Netherlands found that more than half of all interviewed migrant GBV victims experienced direct or peer victimization of violent sexual assaults such as rape, sexual abuse or harassment since arrival in the EU.

Migrants face a high risk of victimization when transiting through third (or subsequent) countries. Female asylum-seekers heading towards the EU during the 2015 EU refugee crisis through the Mediterranean route faced heightened threats of GBV during transit. These were usually in the form of forced transactional sex, coercion by smugglers and/or coastguard, and while in detention in Turkey [38]. This is similar to findings from the Nissling and Murphy-Teixidor [39] survey of over 5000 migrants in Libya stating that women are significantly more likely to experience sexual violence than men in Libya. A study on migrant women in a Sexual Violence Relief centre in Turin, Italy, found that 58.8% of respondents experienced violence while in Libya [40]. In interviews with 72 migrants at an asylum reception centre in France, Reques, Aranda-Fernandez [35] found that 53% of female respondents and 18% of male respondents reported experiencing sexual violence in Libya.

These findings on experiences of GBV while in transit, and in particular Libya, are relatively unsurprising in light of existing reports and articles on the subject. Migrants that travel without legal documentation are vulnerable to multiple types of exploitation and abuse. At the same time, the synthesis stresses the high prevalence of GBV for women in transit and the need to provide further options for protection for this group.

A 2011 IRC rapid assessment showed changes in type of GBV from sexual assault and rape to Intimate Partner Violence (IPV) as refugees moved from Syria to Lebanon [41]. This provides important evidence on how GBV may change forms at different stages of the migration journey as migrants are exposed to different vulnerabilities. As will be elaborated in the next section, findings of this study suggests that differences in authority, (and thus, power), have a direct impact on migrants’ risk of victimization. The lack of documentation and / or financial means to travel in the desired manner contributes to and widens the power potential perpetrators hold over migrants.

### Emerging focus on male victims

Although this field of study traditionally focuses on female victims, research on male victims has emerged in recent years. Studies largely show that in some migratory contexts, GBV perpetration against males and boys can equal that of females and girls. UNHCR reports of widespread GBV against adolescent boys and men in Lebanon and Jordan at rates similar or higher to women [42]. In Jordan, a 2013 inter-agency UN assessment found that refugee boys are perceived to be of higher risk for sexual violence as compared to refugee girls [43]. The socially ascribed values to girls’ sexuality and virginity creates the perception that the rape of boys is less harmful. Parents are also more attentive to girls’ whereabouts as compared to boys – making young boys a more accessible target for predators (Chynoweth, 2017 p.30) [44, 45]..

Adult males are also at-risk of GBV victimization, especially within the migratory context. A 2020 study using MSF clinic data from seven African countries found that more than one-third of all male victims experienced sexual violence within migratory contexts [2]. An IOM report [46] based on a survey conducted with approximately 12,000 migrants who arrived in Italy through the Mediterranean from 2016 to 2018 suggested that male respondents and those between 14 and 24 years old are more likely to have experienced some form of abuse, exploitation, or human trafficking (p.197). Thus, the emerging attention on males and comparative studies between genders have highlighted that males – especially younger males – are also at high risk for GBV perpetration. It is therefore essential to not overlook boys and males in GBV screening and future research.

## Power imbalance as a primary risk-factor

Across the studies, perpetrators of migrant GBV tend to be those that hold some form of authority over the victim, showing the involvement of unequal power dynamics. Smugglers aiding in the travel, authorities such as border guards and/or local police, and where applicable, soldiers of armed groups in conflict or employers [26, 40, 47–51]. Some studies also suggest that locals can be perpetrators in the context of unaccompanied minors, asylum-seekers and refugees [52]. Keygnaert et a.l [37] found that within the context of asylum-seeker arrivals into EU reception centers, the most common perpetrators of GBV are (ex-)partners and asylum professionals.^3^ Delving more into IPV, female irregular migrants, asylum-seekers, and rural-to-urban households seem particularly vulnerable to IPV due to the stresses induced by migration on the perpetrators [32, 53–56].

### Economic hardship as a source of power imbalance

Studies raise the saliency of economic hardship of the victims in exposure to GBV, demonstrating an unequal relationship between victims and perpetrators in control of (access to) resources. Other than vulnerability to being trafficked for sexual exploitation, lack of income-generating activities or lack of the right to work create vulnerabilities when migrants of both sexes have to resort to transactional sex as a survival strategy [26, 37, 57, 58]. Living in insecure housing such as tents or co-ed asylum facilities also increases the risks of GBV for female migrants [3, 59], while the lack of secure transport to places of work can also expose migrants to risk of GBV [60]. GBV may also be used to maintain or express power in times of economic hardship - men’s frustration over their inability to provide for the household, and disruption of traditional gender roles when women enter the workforce to provide or when women are registered as head of households as beneficiaries, may contribute to IPV and/or GBV perpetration [41, 61–63].

### (Lack of) documentation as a source of power imbalance

While maintaining an undocumented status posits high risks to GBV on its own, female migrants tend to face different risks compared to male migrants. In transit, females who travel with smugglers tend to be exposed to heightened risks of GBV and transactional sex [38, 64]. Meyer, Robinson [64] suggested from a study in Thailand that undocumented females are significantly more at risk of GBV with one in three having experienced unwanted sex – with males experiencing more non-sexual physical violence. Border crossings, roadsides, and prison / detention centers are common areas where risks of GBV victimization are higher [52, 65], sometimes to the extent where transactional sex was normalized and ‘expected’ from females [66]. While female migrants face higher rates of transactional and compelled sex, GBV that occurs towards undocumented male migrants tend to be more physically violent [52, 64, 67, 68]. In facing difficulties such as GBV abuse and injuries during travel, men are less likely to be given help and more likely to be left alone [69].

### Rights from national legislations

National legislations regarding the rights of irregular migrants, asylum-seekers, and refugees, can also expose migrants to higher risks of GBV. Fear of deportation or identification due to securitization policies can make migrants more vulnerable to GBV victimization [5], being a threat that potential perpetrators can leverage on. For example, Briddick [70] argued that sponsorship-based foreign partner visas in the EU risks third-country nationals enduring GBV without reporting. Provisional statuses for asylum-seekers or refugee status that do not allow formal employment can also drive migrants into economic hardship, increasing their vulnerability to sexual exploitation and violence [65]. Fear of deportation by losing employment status, partner, or residential status also posits a large barrier to receiving post-GBV care [71]. National legislation and laws are thus risk-factors to GBV victimization when it creates inequalities that put migrants in yielding positions.

## Lasting consequences and fear of disclosure

Migrants are a significant sub-group among those that seek post-GBV care, with studies finding that up to a-fifth of those in Belgium GBV clinics are migrants [72]. Particularly for females, unwanted pregnancies and gynecological health are usually focused on as a push factor for self-disclosure and seeking help. Bronsino, Castagneri [73] suggests that asylum-seekers in Italy between 2016 to 2017 who have reported GBV encounters are more likely to be pregnant and to ask for abortion. In addition, Castagna, Ricciardelli [40] found that more than a-third of migrant females in rape centers in Italy has lasting physical scars after the rape encounter. Pannetier, Ravalihasy [3], using the 2013 Parcours suvey of SSA migrants in Paris, also suggests that social hardship and sexual violence while abroad is strongly associated with situations of HIV aquisition.

Since GBV occurrence to males are usually more violent and used as a form of humiliation or torture [52, 74], health consequences can be more devastating. The qualitative study by Chynoweth, Buscher [52] narrates the extent of physical damage on male migrant GBV victims across refugees from Myanmar, DRC, Somalia, and South Sudan – physical damage often affected male migrants’ ability to socialize and work such as in the case of fecal incontinence. In addition, cultural expectations of male invulnerability posited huge barriers for accessing post-violence care [2]. Since most post-violence clinics may be adapted to female-specific needs, and promoted towards females, males victims have less points of access into care [2].

## Differential understandings of victimhood

Conceptualization and understanding of GBV victimhood differ across stakeholders. Care-providers and international organisations define GBV victimization differently from the communities and victims involved. GBV may be normalized or even expected by victims within certain contexts, to the extent that victims do not consider themselves to be victims. Furthermore, the definition of victimhood should be expanded in order to encompass forced exposure to GBV whom often also struggle with distress and lasting psychological effects.

### Between organisations and communities

Hough [75] argued that migrants’ conceptualization of GBV may be largely different from those of international organisations’ – blurring the lines on whether some victims actually consider themselves as victims. The author elaborated that the concept of sexual consent does not have clear transferability between cultures – sexual passivity, modest refusal and marital partnership can be sometimes denoted as markers of clear consent. The study by Oliveira, Rosário [76] in EARFs similarly found that SGBV conceptualization of residents differed according to respondents’ time of arrival, host country, and type of accommodation. In terms of GBV protection and migration, this makes it difficult for organizations to engage with victims who do not consider themselves as victims, or to approach perpetrators who do not understand the fault of their actions.

### Normalisation of GBV

In transit, females who travel with smugglers tend to be exposed to heightened risks of GBV and transactional sex [38, 64]. As stated previously, border crossings, roadsides, and prison / detention centers are common areas where risks of GBV victimization are higher [52, 65], sometimes to the extent where transactional sex was normalized and ‘expected’ from females [66]. Normalisation of partner abuse, internalized social norms on the subservient position of women within households, and stress from forced displacement all contribute towards IPV occurrence [36, 54, 61, 64]. The study by Welton-Mitchell, Bujang [36] found that over 80% of both male and female Rohingya refugee respondents agreed that men have a right to punish women. At the same time, qualitative research by Chynoweth, Buscher [52] show that men in transit also experience sexual violence with respondents stating such experiences “are normal”.

### Indirect victims of GBV

A further distressing theme that emerged from the literature was that refugees and asylum-seekers often report forced- witnessing or exposure to GBV and / or being compelled to rape [2, 26, 52, 77, 78]. This may be in combination with personal GBV experiences, but these victims usually do not identify themselves through normative understandings of victimhood and thus are usually subsidiary in GBV studies, targets of anti-GBV campaigns, and post-violence clinics. Palillo (2020) explored the strain on young male migrants that have been exposed to woman being violently raped in Libya and being unable to intervene or help them. The loss of masculinities and feelings of helplessness lead to lasting psychological challenges. More research in this area is warranted as forced exposure and compelled rape can also have heavy psychological consequences for victims.

## Discussions

This review has demonstrated the high prevalence of GBV amongst migrants and the need for further research and programming to assist this vulnerable population. Several key themes have emerged through the synthesis of the 84 studies.

First, GBV experiences often differ across the migration stages, leading to migrants experiencing different forms of GBV at different stages of migration by different perpetrators. It is necessary for further understandings in this area of how GBV experiences change across time and space in the migration experience, and resultantly how support programmes and interventions can be targeted at the different migration stages towards different forms of GBV.

Second, it is clear from the studies that migrants’ experiences of GBV victimization occurs within the context of power imbalances. Common perpetrators are those that hold some form of power over the migrant – for example, border authorities, smugglers, or even locals within host countries. Accordingly, vulnerable migrants that travel without documentation, taking precarious routes, and whom may seek to evade detection by local authorities are at-risk of GBV victimization. In some cases, females even partner up with males when facing GBV risks – engaging in (usually forced) marriages for male protection [32, 41], which should be examined as an attempt to gain power in facing other, potential perpetrators.

Power imbalances in gender-based violence have been well-studied, albeit to a much lesser extent within the field of migration and GBV. Factors such as the lack of documentation and financial means reduces the ability of migrants to firstly, seek redress for any injustice. The inability to report on GBV experiences or to seek formal medical help without detection of irregular status or stigma allows for potential subjugation of the migrants with little to no real consequences. Secondly, traveling with little legal and / or financial means forms a reliance on the goodwill and discretion of those surrounding the migrants. Just as border guards may rely on their discretion to allow a migrant through, locals, smugglers, or even other migrants can report an irregular migrant over a small dispute. The reliance on others’ discretion places migrants in a submissive position with little authority and control over their own trajectories. This widening disparity of power undeniably increases migrants’ risk of GBV victimization.

Third, both emotional and physical coping mechanisms for GBV victims are largely internalized – meaning that victims often do not seek help unless there are acute physical concerns that need to be addressed. Most migrants fear the stigmatizations attached to being a victim of GBV and therefore do not disclose their experiences. In particular, male victims are less likely to seek help. This means that the number of male GBV experiences are likely to be severely under-reported and consequently, under-researched as compared to female GBV experiences.

This leads to the fourth key point that there is a need for further research on male migrants’ experiences of GBV, as a victim, partner, and through other forms of exposure. There are few studies comparing GBV characteristics between both genders, as most studies are focused on either females or males. While it may be likely that female migrants face a higher risk of GBV as normatively understood, male GBV victims may experience more mental and social distress, barriers to receiving post-GBV care, and barriers to disclosure [52]. Other studies also suggest that GBV acts are associated with access to females [6] (lower access can transfer violence onto males) and humanitarian focus on females that neglect males [79]. More comparisons of GBV characteristics between genders and sexes can provide insights into how sexual violence can be displaced from one gender and sex to another. Such insights can also help to substantiate a critical discourse on the *gender* aspect of gender-based violence. There needs to be a clearer delineation between first, the relationship between genders within the field of GBV, and second, how gender is perceived by perpetrators, victims, and relevant third-parties. Such conceptualizations have a strong potential in framing future GBV prevention policies.

Fifth, the studies show that the understanding and conceptualization of GBV differ across stakeholders. In particular, care-providers and victims may differ in their understanding of victimhood and GBV acts. Notions of victimhood may also be challenged in cases where transactional or survival sex is normalized or expected, blurring the lines between victims’ understanding of forced or voluntary participation. Furthermore, victims of GBV often also include those who experienced forced-witnessing and forced-participation, despite not being the target of GBV acts themselves. There is a lack of research on these indirect victims and the consequences of their forced experiences. This brings forward the question of how to include indirect victims in research and programme support.

Lastly, the focus on females within GBV prevention and policies not only marginalizes male victims, but also deny males an active role to play in prevention. Across frameworks and policies, the marginalization of male migrant GBV prevention and support has been consistently raised in the literature [2, 52, 67, 80]. This neglect was also replicated in policy evaluations and academic research - Kiss, Quinlan-Davidson [81] argued in a literature review that most intervention evaluations for GBV survivors do not provide male-specific components. This is despite multiple studies showing marked differences in GBV victim characteristics between males and females – males victims are more likely to present themselves to healthcare support when GBV disclosure is not necessary [2], face more undisclosed social stigma and risk continued victimization if identified as GBV victim [52] [82]. Targeting the root of power imbalances within community- or male-targeted interventions can potentially play a strong role in gender-inclusive GBV prevention.

### Research and literature limitations

Due to the researchers’ language barriers, the study was unable to include non-English papers for review. This results in possible over-representation of migrants living or traveling towards English-speaking regions in our study. Moreover, the current labelling of types of gender-violence is not exhaustive – there are other types of experiences of GBV, such as witnessing and other forms of exposure, either to the acts, victims, or perpetrators. Since the search and review process was guided by existing terms, this study does not wholly represent all types of GBV. Further research on types of GBV and the sufficiency of current terms used in identification, prevention and protection should be encouraged.

In addition to the key points raised above from the inductive CIS approach, this review has also identified several limitations within the existing literature. First, topics of resilience were not frequently mentioned in literature. This is important in understanding the longer-term effects of GBV on migrants’ mental health and future life satisfaction and opportunities. This highlights that further research on how migrants cope with the aftermath of GBV and whether a singular incidence of GBV exposes the migrant to subsequent risks should be explored.

Second, within the discourse on the nature of GBV acts, only four studies compared experiences between pre-migration exposure to GBV and GBV incidences after movement [56, 64, 83, 84]. These studies compared levels of GBV vulnerabilities faced in origin and destination countries, usually within the context of viewing conflict-related violence as part of migrants’ GBV experiences [64, 84]. Further, only seven studies explored GBV prevalence and vulnerabilities during transit [5, 40, 47, 52, 66, 69, 73], with one study being conducted in a transit country [47]. There is a need for further research in both these areas in order to more comprehensively understand the extent of vulnerabilities faced by migrants throughout different parts of their journey.

Third, echoing the findings from the literature, in addition to insufficient research on male experiences of GBV, there is a dearth of literature on LGBTQI migrants’ experiences within the field of GBV. As evidenced in other areas of migration research LGBTQI migrants often face gender specific forms of discrimination and violence, suggesting the need for further examination of LGBTQI migrants’ experiences of GBV.

### Policy and programme consideration

Finally, the review has identified two important areas for policy and programme considerations. First, a central finding in this review is that experiences of GBV were recorded the most amongst forced migrants and undocumented migrants, wherein during the migration journey migrants were at high risk of GBV. This stresses the importance of gender sensitive reception policies that are able to identify and support victims of GBV upon arrival to the EU or another host country. Within the EU context it is difficult to prevent GBV in countries such as Libya, wherein there has been an absence of state control and authority. However, gender sensitive reception approaches that takes into account differences in experiences and protection needs can better identify and provide care for victims [85].

A second consideration for policy and programming is the need for creating consensus in definitions and approaches to GBV that can enable a consolidation of GBV data among migration-related institutions such as UNHCR and IOM. This is necessary in order to establish clearer and comparative insights on how, when, and where migrants encounter GBV. Although some microdata currently exists on GBV among migrants, they are largely mixed with other migrant protection indicators. Isolating and therefore retrieval of GBV-focused data is difficult. Standardization of methodology such as on sampling methods and how survey or interview questions are asked (direct vs. indirect questions) can further support research in this field.

Further to this, academic research also needs to find alignment in terminology within the field of GBV. Given the regions of prevalence wherein migrants experience GBV it is important to have transnational cooperation in research in this field.

## Conclusion

This critical interpretive synthesis sought to understand worldwide migrant experiences of gender-based violence. With an inclusion-criteria for literature search that focused on migrant experiences of gender-based violence, 67 peer-reviewed academic articles and 17 documents of “grey” literature were reviewed and synthesized. The results allowed us to form certain conclusions to inform future research. Syntheses of existing papers show that firstly, most GBV occurrences are rooted in power imbalances, second, have lasting consequences on victims, and third, that there are different understandings and conceptualizations of GBV victimhood across stakeholders. Overall, research on GBV is still primarily focused on prevalence reporting and consequences, especially on GBV occurrences in destination countries. Further research focusing on GBV experiences throughout migrants’ journey, coping mechanisms, and male experiences of GBV should be encouraged.

This review has identified several potential areas for future research including:

1. The need for high quality discourse on how power imbalances increase GBV risks specific to the field of migration.
2. The need to conduct high quality studies on effective approaches to prevent GBV within migrant populations covering their stay and transit.
3. The need to conduct high quality studies on providing effective support to victims of GBV during their migration journeys covering their stay and transit.
4. Sensitive longitudinal research to understand the lasting impacts of GBV experiences on migrant’s mental health.
5. The need for a culturally sensitive standardized tool to assess prevalence, severity, and continuation of GBV experiences over time. This will not only help future policy planning, but also to have a clearer understanding on the types and severity of GBV encountered by migrants as they move from one region to the next.
6. Testing of best methods for collecting data on GBV is necessary to ensure protection of migrants while also being able to provide more information for policy makers and practitioners on this sensitive topic.

## Data Availability

NA

## Abbreviations

API-GBV: Asian-Pacific Institute on Gender-Based Violence
ASIST-GBV: Assessment Screen to Identify Survivors Toolkit for Gender Based Violence
CIS: Critical Interpretive Synthesis
CBPR: Community-based Participatory Research
EU FRA: European Union Fundamental Rights Agency
WoS: WebofScience
GBV: Gender-Based Violence
UNHCR: United Nations High Commissioner for Refugees
IDP: Internally Displaced Person
ILO: International Labor Organisation
IPV: Intimate Partner Violence
IOM: International Organisation for Migration
MENA: Middle East and Northern Africa
MSF: Médecins Sans Frontières
IFRC: International Federation of Red Cross
EU: European Union
SSA: Sub-Saharan Africa
NGO: Non-governmental Organisation
UNICEF: United Nations International Children’s Emergency Fund
UN ESCAP: United Nations Economic and Social Commission for Asia and the Pacific
UNODC: United Nations Office on Drugs and Crime
MICS: Multiple Indicator Cluster Surveys

## Footnotes

1 Launched on 3rd March 2021. As of the first submission date of this article, it only consists of COVID19 migration-related data until further updates to include more data.

2 Belgium, Greece, Hungary, Ireland, Malta, The Netherlands, and Portugal

3 Referring to professional service provider staff working for and from asylum centres.
